# Soil microbial growth rate, trophic strategy, and the oligotroph–copiotroph continuum

**DOI:** 10.1128/aem.01324-26

**Published:** 2026-08-10

**Authors:** Chansotheary Dang, Juan Piñeiro Nevado, Ronald Schartiger, Binu M. Tripathi, Jeth G. Walkup, Edward Brzostek, Ember M. Morrissey

**Affiliations:** 1 Department of Biology, West Virginia University 240297, Morgantown, West Virginia, USA; 2 Division of Plant and Soil Sciences, West Virginia University 5631, Morgantown, West Virginia, USA; 3 School of Forest Engineering and Natural Resources, Polytechnic University of Madrid 16771, Madrid, Spain; 4 Department of Ecology and Evolutionary Biology, University of California 189205, Irvine, California, USA; Georgia Institute of Technology, Atlanta, Georgia, USA

**Keywords:** microbial growth rates, oligotroph-copiotroph continuum, life-history strategy, trophic strategy, quantitative stable isotope probing, soil habitat, rhizosphere

## Abstract

The oligotroph–copiotroph continuum is an ecological framework that describes microbial metabolic traits, habitat preferences, and growth strategies. As microbial growth rates determine soil element cycling, understanding microbial trophic strategies is critical for improving predictions of ecosystem biogeochemistry. However, accurately determining microbial trophic strategies has been challenging for soil microorganisms. Here, we examined microbial growth rates (via ^18^O-quantitative stable isotope probing) in rhizosphere and bulk forest soils and proposed a new approach to determine the trophic strategies of microbial taxa. Growth rates were log-normally distributed and used to classify 35% of bacterial taxa as oligotrophs (lowest growth rates), 50% as mesotrophs (intermediate growth rates), and 15% as copiotrophs (highest growth rates). The average growth rates of individual taxa exhibited phylogenetic organization, suggesting that growth rates are constrained by vertically transmitted genomic traits under varying conditions. Consistent with the expected habitat preferences, copiotrophs were more abundant and active in the rhizosphere, while bulk soil was dominated by slow-growing oligotrophs. Carbon addition positively impacted copiotroph growth rates in both the rhizosphere and bulk soil. Oligotrophic and mesotrophic taxa benefited from carbon addition in the bulk soil, but not in the rhizosphere, perhaps due to intensified competition with a large population of copiotrophs. Taken together, these results demonstrate that microbial growth rates are tied to bacterial habitat preferences and carbon responses, suggesting they may be strong indicators of trophic strategy.

**IMPORTANCE:**

Despite compelling culture-based evidence for the oligotroph–copiotroph framework, scientists have struggled for decades to meaningfully characterize the trophic strategies of soil microorganisms, perhaps due to methodological limitations. This work uses a growth rate approach to determine the trophic strategies of microbial taxa that is validated by testing two classic hypotheses regarding microbial trophic strategy in soil. The results support the use of growth rate–based trophic strategy classification and demonstrate that microbial growth rates are constrained by evolutionary history such that fine (e.g., families and genera) but not coarse (e.g., phyla and orders) taxonomic groups may be meaningfully described as oligotrophs or copiotrophs. These results are of broad relevance, as they suggest that growth rate measurements may be used to make a prevailing ecological theory describing all unicellular microbial life on Earth (i.e., the oligotroph–copiotroph framework) more quantitative and robust.

## INTRODUCTION

Biogeochemical cycles and nutrient transformation are heavily regulated by the activities of heterotrophic microorganisms in soil (1, 2). Surface soil microbial communities are dominated by heterotrophic bacteria and fungi, and the activities of these taxa determine the rates of organic matter decomposition and ultimately influence whole ecosystem carbon balance (3). However, determining the impact of individual taxa on broadly distributed functions, such as soil organic matter decomposition, remains challenging due to the phylogenetic and metabolic diversity of the organisms involved.

Trait-based ecological strategy frameworks describe how organisms allocate resources to growth, resource acquisition, and survival under various environmental conditions and can be used to understand an organism’s function within an ecosystem (e.g., r/K selection theory [4], the competitor–stress-tolerator–ruderal framework [5], the yield–acquisition–stress tolerance framework [6]). Ecological strategies arise from eco-evolutionary trade-offs that influence population dynamics, competitive interactions, and ecosystem-level processes (as reviewed in references 7–10). A widely used framework in microbial ecology is the oligotroph–copiotroph continuum, developed from pure culture studies describing habitats and growth rates of bacteria. This framework was later applied in microbial ecology to classify the trophic strategies of soil heterotrophs (11, 12). Here, we use microbial “trophic strategy” in the context of the oligotroph–copiotroph framework to refer to a suite of traits that influence an organism’s resource acquisition in the context of ecological competition. Oligotrophic microbes are adapted to nutrient-poor environments, exhibit high substrate affinity, slow growth rates, and high growth efficiency, while copiotrophic microbes thrive in resource-rich conditions, have rapid growth, larger genome sizes, and enhanced extracellular enzyme capabilities (13). Due to the physiological differences between copiotrophs and oligotrophs, the ratio of copiotrophs-to-oligotrophs is posited to influence community-level microbial function (14). However, strong quantitative connections between microbial trophic strategies and ecosystem process rates remain scarce (15, 16). Slow progress in this area could be due, at least in part, to inaccurate and imprecise placement of microbial taxa along the oligotroph–copiotroph continuum.

The imprecise placement of microbes may be fueled by the overreliance on coarse taxonomic groups, which fail to account for substantial physiological variation within taxa of the same phylum. Studies frequently designate entire phyla as either oligotrophic or copiotrophic, often yielding conflicting findings. For example, Proteobacteria, Actinobacteriota, and Firmicutes have been classified as copiotrophs in some studies (12, 17, 18) and oligotrophs in others (19, 20). Classifying trophic strategies at a finer taxonomic level (e.g., genera or families) may be more reliable (20, 21). Additionally, in soil, trophic strategy is largely determined by changes in relative abundance of 16S rRNA genes in response to carbon and nutrient amendments (for a review, see reference 22). Relative abundance data are inherently non-independent, wherein changes in the abundance of one taxon impact the relative abundance of all others. This interdependence precludes confident determination of increases and decreases in population size (23).

Although early descriptions of microbial traits related to oligotrophy describe a continuous spectrum (24), the oligotroph–copiotroph framework has been largely discussed as a dichotomy (e.g., see references 11, 12, 25), and microbes are typically classified into one of two groups. However, many microorganisms exhibit intermediate growth rates (21, 26) and may be better described as mesotrophs. An organism’s growth rate is an integrative measure of survival, resource assimilation, and biomass production (27); as such, growth can be a strong indicator of trophic strategy. Additionally, microbial growth rate can now be measured as a continuous variable in natural communities via quantitative stable isotope probing (qSIP) (28, 29). As such, it is possible to examine the distribution of growth rates and achieve a more precise placement of individual microbial taxa along the continuum from oligotroph to copiotroph.

Microbial trophic strategy plays a fundamental role in shaping microbial habitat preferences, as consistently demonstrated by numerous studies (11, 13, 18). This is most evident in aquatic environments, with nutrient-poor environments harboring slow-growing oligotrophs, and nutrient-rich environments being dominated by fast-growing copiotrophs (30, 31). Soil environments are more heterogeneous and contain distinct microhabitats due to spatial variation in resource availability. For instance, the rhizosphere, enriched with labile carbon from root exudates, has been posited to favor copiotrophs, while bulk soil, with infrequent and complex carbon inputs, is argued to select for more oligotrophic microbes (32, 33).

Here, we used bacterial growth rates to assess the trophic strategies of soil bacteria along the oligotroph–copiotroph continuum. While bacterial growth rates and trophic strategies exist on a continuous spectrum, accompanying trophic classifications can assist in hypothesis generation and testing, and improve our ability to understand, describe, and communicate natural patterns in microbial ecology. To assess the validity of growth rate–based trophic strategy assessments, we tested two classic hypotheses associated with the oligotroph–copiotroph framework in microbial ecology. Hypothesis 1: fast-growing copiotrophs prefer resource-rich habitats and thus will be more abundant and active in the rhizosphere, whereas slow-growing oligotrophs prefer resource-poor environments and thus will dominate the bulk soil habitat. Hypothesis 2: high-resource availability benefits fast-growing copiotrophs but not slow-growing oligotrophs, and thus labile carbon will only increase the growth rate and relative abundance of copiotrophic taxa. Since microbial trophic strategies are shaped by evolutionary processes, we further predicted that growth rate exhibits a phylogenetic signal, and closely related taxa have similar growth rates. Phylogenetic patterns in bacterial growth provide a foundation for classifying bacterial taxa as oligotrophs, mesotrophs, or copiotrophs. Microbial growth rates were measured via ^18^O-qSIP in rhizosphere and bulk soil habitats collected surrounding arbuscular mycorrhizal tree species in a temperate forest. A gradient of synthetic root exudate amendments (0, 250, 500, and 1,000 µg-C g-soil^−1^) was used to simulate the labile carbon typically found in the rhizosphere and assess changes in taxon-specific growth rate and relative abundance in response to carbon addition. This work describes a new approach to assess the trophic strategies of bacterial taxa using growth rates to more accurately describe the ecology of microbes along the oligotroph–copiotroph continuum.

## MATERIALS AND METHODS

### Soil sampling and isotope incubation

Soils were collected from the Fernow Experimental Forest watershed 4 (39.05, −79.69) located in Parsons, WV, USA, in July 2020 as described for the arbuscular mycorrhizal plots in Tripathi et al. (34). Briefly, we sampled soil from five plots (25 × 25 m) within a 0.03 km^2^ area. Within each plot, nine soil cores (5 cm diameter × 10 cm depth) were collected from around the perimeter of three arbuscular mycorrhizal (AM) trees (*Acer rubrum, Acer saccharum,* and *Liriodendron*). Rhizosphere and bulk soil were separated onsite by hand-shaking live roots (35), and rhizosphere and bulk soil were composited for each plot. Samples were transported on ice and stored at 4°C in the laboratory overnight. Soils were sieved (2 mm) and dried for 48 h at room temperature to reduce soil moisture to an average of 19% to 7% gravimetric water content to facilitate H_2_^18^O addition (36); water holding capacity (WHC) was determined as described in Walkup et al. (37).

Approximately 72 h after sample collection, time zero (*t*_0_) samples were frozen for DNA extraction and used to calculate the natural abundance weighted average density (unlabeled samples, *n* = 10 total), and the isotope incubation experiment was initiated. Subsamples (2 g) of dried rhizosphere and bulk soil from each plot were adjusted to 60% WHC with 97 atom% H_2_^18^O without carbon addition or with 250, 500, or 1,000 µg-C g-soil^−1^. These concentrations were chosen to reflect a wide range of exudation rates observed in AM trees (38). Root exudate solution contained a mix of sugars and organic acids described in Dang and Morrissey (39). Samples were incubated at 23°C for 5 days and then stored at −20°C prior to molecular analysis (*t*_5_). In total, the experiment had two soil habitats (rhizosphere and bulk) and four substrate treatments, each with soils from five replicate plots (40 total *t*_5_ samples, labeled with ^18^O-H_2_O). Parallel microcosm incubations with identical treatments and moisture levels were used to measure total soil respiration. Briefly, 12 g (dry weight equivalent) of soil subsamples was sealed in 950 mL mason jars and adjusted to 60% WHC with water or exudate solution. Total CO_2_ in the headspace of each microcosm was measured on days 1, 3, and 5 using a LI-6400XT (LI-COR Biosciences, Lincoln, NE, USA), similar to Walkup et al. (37).

### DNA extractions and quantitative stable isotope probing

An internal standard of *Halobacterium salinarum* genomic DNA (20 ng) was added to approximately 0.25 g of soil (40) before DNA extraction. Total DNA was extracted using the Qiagen DNeasy Power Soil kit, and DNA concentration was quantified with the Qubit 4 Fluorometer. Taxon-specific growth was measured using ^18^O-water qSIP (28) as described in reference 34. Briefly, 1,000–2,000 ng of DNA was separated by isopycnic centrifugation over 72 h in an Optima Max ultracentrifuge. DNA fractions (~145–150 µL) were collected and purified with isopropanol and ethanol before resuspension in PCR water. DNA in the density range of 1.6353–1.7209 g·mL^−1^ was quantified via quantitative PCR (qPCR) for 16S rRNA gene abundance as in reference 34.

### DNA sequencing and bioinformatics

The V4 hypervariable region of the prokaryotic 16S rRNA gene was sequenced for whole community DNA extracts (unfractionated) and qSIP fractionated DNA samples using an Illumina MiSeq (2 × 250 bp paired-end reads) with dual-indexed primers (515f and 806r) as described in Kozich et al. (41) (Michigan State University, Genomic Core). In QIIME 2 (version 2021.2), paired-end reads were joined using the “qiime vsearch join-pairs” function. Denoising was performed separately for each Illumina MiSeq run using the q2-deblur plugin, trimmed at 250 bp, and then all feature tables and data were merged. Taxonomic assignment was performed with the “q2-feature classifiers” plugin with the RESCRIPt SILVA 138 16S rRNA gene reference database (99% majority sequencing identity) (42, 43), and chloroplast and mitochondrial sequences were removed. The feature table was exported from QIIME 2 at the ASV level. For qSIP and all downstream statistical analyses, ASV read counts were aggregated by species, and all analyses were conducted at the species level. Taxa with a frequency of <0.5% of the mean total frequency (<985 total sequence count) were removed (~0.5%–5.7 % 16S rRNA genes per sample).

### Taxon-specific growth rates with ^18^O-enrichment

Growth rates were determined by calculating the shift in weighted average densities (WAD) of every taxon (*t*_5_-labeled − *t*_0_-unlabeled) following ^18^O assimilation into DNA (28, 29). The density of the internal standard (*H. salinarum*) was used to correct for analytical imprecision introduced during ultracentrifugation as described in reference 34. To ensure robust growth rate estimations, taxa present in less than 4 *t*_0_ replicates and taxa with unreliable enrichment estimates (mean standard deviation >0.005) were removed. Approximately 90% and 92% of the *t*_0_ community were retained for bulk and rhizosphere soil, respectively. Excess atom fractions (EAFs) were calculated according to Hungate et al. (28), and negative EAF values were converted to zero. Only taxa present in the whole community (unfractionated) samples were considered for further analysis (348 total taxa, which accounted for 79%–93% 16S rRNA genes per sample). Taxon-specific relative growth rates (RGR) per day were calculated as follows: EAF_(taxon i)_ × [^18^O-enrichment_(soil j)_ × 0.6 × 5 days]^−1^, with 0.6 representing the maximum proportion of oxygen atoms in DNA derived from water and 5 days being the incubation length (44, 45). The community RGR was calculated by averaging the RGR of each taxon within each replicate, and active taxa are those with an RGR above zero.

### Statistical analysis

Statistical analysis was performed in R (version 4.2.3) (46) with α = 0.05. Alpha and beta diversity metrics were calculated using the “vegan” package. A two-way PerMANOVA (soil habitat × carbon amendment) and nonmetric multidimensional analysis based on Bray–Curtis dissimilarities with Hellinger-transformed abundance data were used to visualize microbial community composition. Treatment effects on alpha diversity and soil respiration were determined by two-way ANOVA (soil habitat × carbon amendment). Variation in RGRs in response to soil habitat and carbon addition was assessed via linear mixed modeling with taxon as a random effect [RGR ~ soil habitat × carbon addition + (1 | taxonID)] using the lmer function (lme4 package, 47). Subsequent variance partitioning (VarCorr function) was used to determine within vs between taxon variation. Spearman’s Rank correlation was used to assess consistency in the RGRs of taxa across carbon addition levels. Differences in taxon-specific RGR between soil habitats and across carbon amendments (within a habitat) were determined via Kruskal-Wallis tests. While bacterial growth rates and trophic strategies exist on a continuous spectrum, categorical trophic classifications were used to facilitate hypothesis testing. As such, a kernel density plot of mean taxon-specific RGR was generated to identify thresholds for trophic classification (oligotroph, mesotroph, and copiotroph); this plot revealed a roughly log-normal distribution with a skew to the right. Trophic strategy thresholds were selected to coincide with the peak of the distribution (oligotroph–mesotroph) and the right shoulder (mesotroph–copiotroph) as described in the Results. Taxa with mean RGRs below the peak were classified as oligotrophs, taxa with RGRs skewed to the right were copiotrophs, and those with values between the peak midpoint but not skewed to the right were classified as mesotrophs. Differences in relative abundance across trophic strategies, soil habitats, and carbon additions were determined using a three-way ANOVA. Effect sizes for factorial ANOVA were estimated using partial eta-squared (η^2^p). For Kruskal–Wallis tests, eta-squared was used to estimate the effect size of carbon amendments (within soil habitat) and between soil habitats. To assess the effect of C addition on taxa within each trophic group, the change in growth between no carbon addition and the highly carbon addition level (1,000 µg-C g-soil^−1^) was calculated as ΔRGR = mean RGR_1,000_ − mean RGR_0_. To assess whether the distribution of ΔRGR was significantly different from 0, the Wilcoxon signed-rank test (rstatix package [48]) was used. Within each soil habitat, the percentage of taxa with increased growth was calculated as the proportion with ΔRGR >0. For phylogenetic analysis, a consensus sequence was generated for each species with the “DECIPHER” package using ASV sequences from QIIME 2 output (49). Sequence alignment and phylogenetic tree were constructed with SINA multiple sequence alignment (50). Briefly, the phylogenetic tree was computed by inserting 16S rRNA partial gene sequences into the nearest neighbor tree (de novo tree generated using full-length sequences of the 10 nearest neighbors for each sequence). The phylogenetic tree was imported into R and pruned to contain only the taxa present in our soils. The “phytools” package was used to calculate Pagel’s lambda and ancestral trait estimates (51, 52).

## RESULTS

### Bacterial community composition and soil respiration

Microbial community composition differed between the rhizosphere and bulk soils (Table 1; Fig. S1). While species richness was similar (*F*_Soil_ = 0.11, *P* = 0.75, η^2^p = 0.003), the microbial community in the bulk soil was more evenly distributed as indicated by the higher Pielou’s Evenness (*F*_Soil_ = 10.5, *P* = 0.003, η^2^p = 0.25) and Shannon’s Index (*F*_Soil_ = 7.10, *P* = 0.01, η^2^p = 0.18). Exudate addition did not alter alpha diversity (two-way ANOVA, all *P* > 0.05). Community composition also differed between the rhizosphere and bulk soil, and changed in response to exudate addition in the rhizosphere soil (two-way PerMANOVA, *F*_Soil × Exudate_ = 1.70, *P* = 0.036, *R*^2^ = 0.07). This can be visualized in the NMDS plot; soil habitats are clustered separately, and the highest level of exudate addition produced shifts in composition in the rhizosphere (Fig. S1). Soil carbon mineralization rate was ~2× greater in the rhizosphere than in bulk soil in the absence of carbon addition. In both soil habitats, increasing carbon addition levels produced consistent and proportional increases in soil respiration (Fig. S2, two-way ANOVA, *F*_Soil_ = 25.7, *F*_Exudate_ = 25.9, both *P* < 0.0001).

**TABLE 1:** Alpha diversity metrics at the community level for bulk and rhizosphere soil habitats (mean ± standard error)^*a*^

Alpha diversity metric	Bulk	Rhizosphere	Two-way ANOVA	*P* value
Species richness	367 ± 5.62	369 ± 4.25	NS	>0.05
Pielou’s evenness	0.78 ± 0.01	0.75 ± 0.01	Soil habitat	0.003
Shannon–Weiner	4.58 ± 0.05	4.43 ± 0.03	Soil habitat	0.01

^*a*^ A two-way ANOVA test was used to assess significant differences (*P* < 0.05) between soil habitat (bulk vs rhizosphere) and root exudates carbon amendment (0, 250, 500, and 1,000 µg-C g-soil^−1^). Since there was no significant effect of carbon addition or interaction, the average for soil habitat across all carbon levels is presented (*n* = 20 per habitat).

### Bacterial relative growth rates and trophic strategies

Taxon-specific RGRs varied in response to soil habitat, carbon addition, and their interaction as determined via linear mixed model analysis (Fig. S3; Table 2). Soil habitat (rhizosphere vs bulk) had the greatest effect (*F*_Soil_ = 213.6, *P* < 0.001), followed by carbon addition (*F* = 59.9, *P* < 0.001), and their interaction (*F* = 41.1, *P* < 0 0.001). The number of taxa with RGR >0 varied across habitats, with 41 found only in the bulk soil, 115 found only in the rhizosphere, and 192 shared across habitats (Fig. S1A and S3). Of the active taxa shared across both soil habitats, ~68% had similar growth rates in both habitats, while ~32% exhibited higher RGR in the rhizosphere (Kruskal–Wallis *P* < 0.05, η^2^ = 0.09–0.70; see Table S2). In contrast to habitat, carbon addition did not significantly alter RGR for 90% of the taxa in the rhizosphere and 99% of the taxa in the bulk soil as determined by Kruskal–Wallis (Fig. S3). Across all habitats and treatments, differences among taxa explained the majority of variation (57%), indicating greater inter- than intra-taxon variation (Table 2). Additionally, RGRs for individual taxa covaried strongly across carbon additions in both the bulk soil (Spearman rho 0.62–0.75) and rhizosphere habitats (Spearman rho 0.67–0.82), indicating consistency in taxon-specific growth ranking (Fig. S5).

**TABLE 2:** Effects of soil habitat and carbon (C) exudate addition on taxon-specific RGR as determined via linear mixed modeling^*a*^

Source	Num df	Den df	F	*P*	Variance (×10^−3^)	% of total
Fixed effects (Type III ANOVA)						
Soil habitat	1	10,584	213.6	<0.001		
Carbon exudate	3	10,361	59.9	<0.001		
Soil habitat × carbon exudate	3	10,361	41.1	<0.001		
Random effects (variance partition)						
Between taxon					0.805	57.0
Within taxon					0.607	43.0
Repeatability (R = ICC)						0.570

^*a*^ Taxon identity was included as a random effect, and variation partition was used to determine intra- vs inter-taxon variation. Repeatability R = Var(taxon) / [Var(taxon) + Var(residual)] is the proportion of growth-rate variance attributable to consistent between-taxon differences (= “% of total” for the between-taxon component). Num df = numerator degrees of freedom, Den df = denominator degrees of freedom.

**Fig 1:**
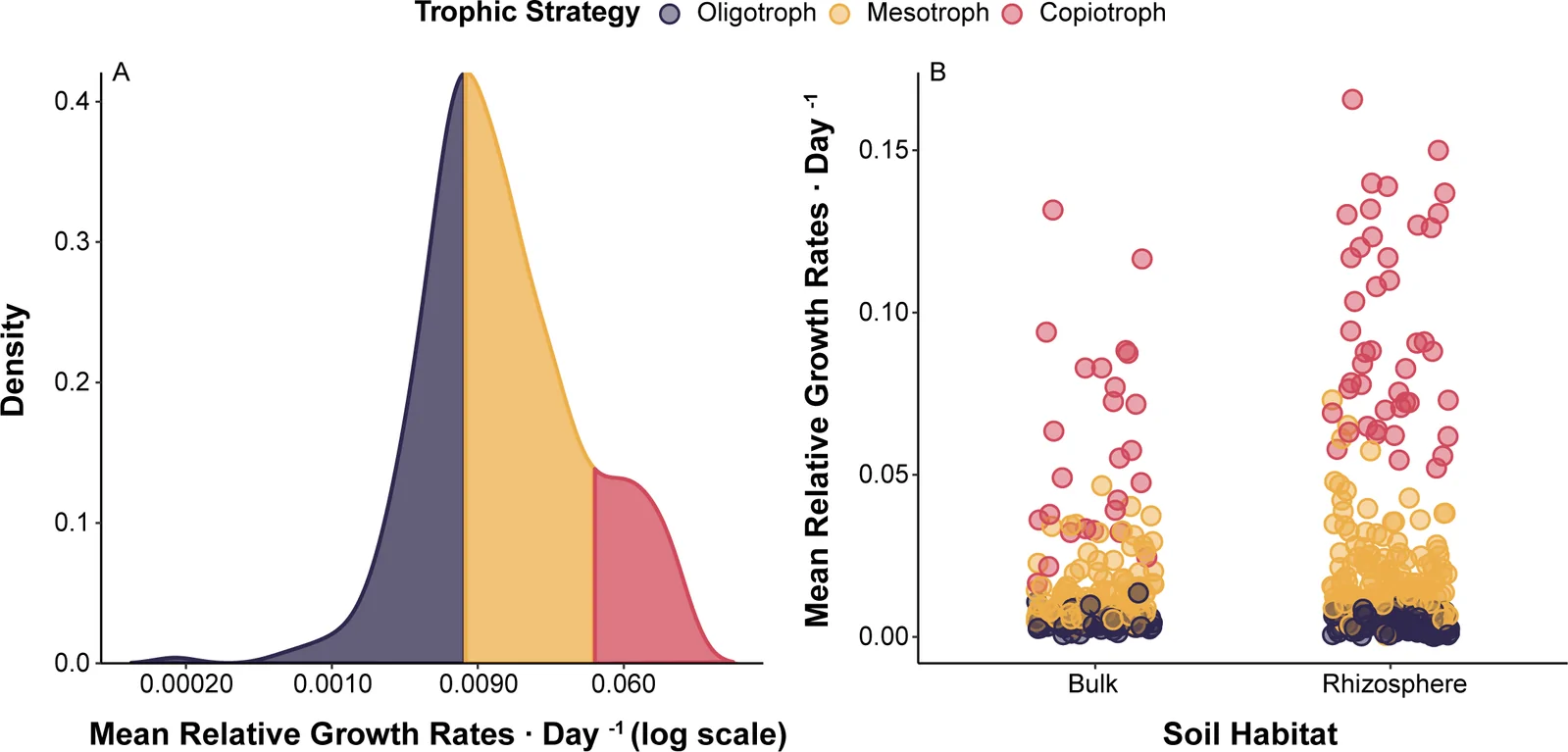
Distribution of taxon-specific mean RGRs. A kernel density estimation of taxon-specific mean RGRs (log-transformed), averaged across both soil habitats (bulk and rhizosphere) and carbon addition treatments (**A**, note log scale on x-axis). Thresholds for trophic strategy classification were defined based on the distribution peak (0.0073) and the beginning of the right skew (0.043). This resulted in oligotrophs being the bottom 35% (≤0.0073), mesotrophs as the middle 50% (>0.0073 and <0.043), and copiotrophs as the top 15% (≥0.043). The mean RGR for each taxon in bulk and rhizosphere soils, averaged across carbon additions (**B**, note linear scale on y-axis). Points colored by trophic strategy based on the overall mean RGR. Some taxa appear above or below the thresholds in specific environments, reflecting condition-dependent variation in growth relative to their overall classification.

To classify taxa as oligotrophic, mesotrophic, or copiotrophic, taxon-specific RGRs were averaged across soil habitats and root exudate addition levels. Growth rates exhibited a roughly log-normal distribution with a skew to the right (Fig. 1A). Trophic strategy thresholds were selected to coincide with the peak of the distribution (oligotroph–mesotroph) and the right-shoulder skewed taxa (mesotroph–copiotroph). Using these criteria, oligotrophs were the bottom 35% (RGR ≤0.0073), mesotrophs were the middle 50% (0.0073 < RGR < 0.043), and copiotrophs were the top 15% (RGR ≥0.043) of taxa in terms of mean RGR. While copiotrophs comprised a comparatively small number of the taxa, these organisms had RGRs that were positively skewed and well above average in both bulk and rhizosphere soils (Fig. 1B).

### Phylogenetic and taxonomic patterns in growth rate and trophic strategy

**Fig 2:**
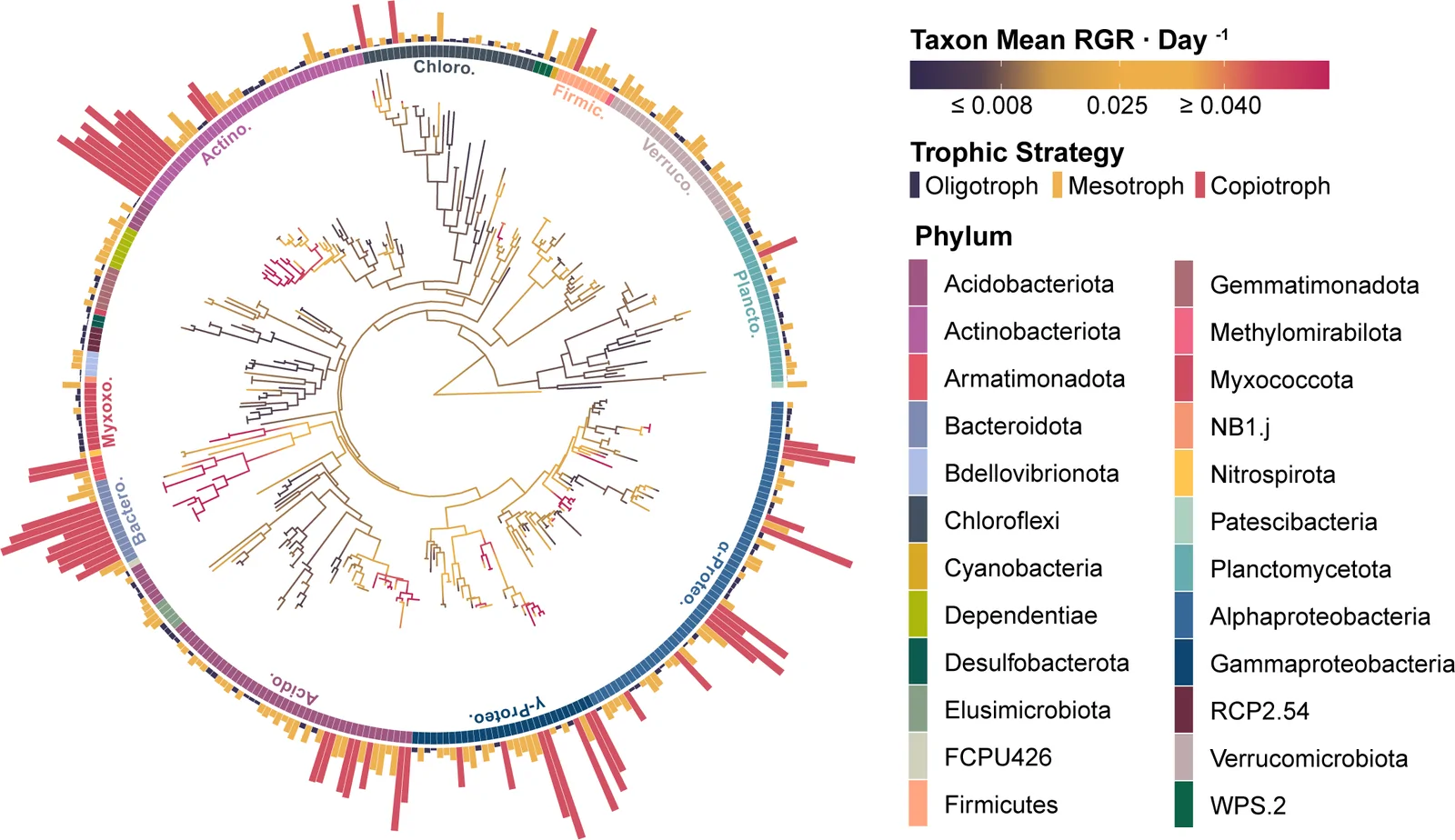
Phylogenetic tree colored by the RGRs of taxa (tips) and ancestral character estimates (nodes and branches) with values indicated on the gradient scale. Bars represent the mean taxon-specific RGRs (averaged across all treatments), ranging from 0.0068 to 0.15, and are colored by trophic strategy (oligotrophs ≤ 0.0073, mesotrophs >0.0073 and <0.043, and copiotrophs ≥0.0043).

The mean RGR of bacteria exhibited a statistically significant phylogenetic signal (Pagel λ = 0.90 and *P* < 0.001). The λ value close to 1.0 indicates that variation in growth rate across the phylogeny is consistent with a Brownian model of evolution and moderate phylogenetic conservation (51). Given the significant phylogenetic signal in microbial RGRs, ancestral character state estimation was used to identify clades likely to be oligotrophic, mesotrophic, and copiotrophic (Fig. 2).

**Fig 3:**
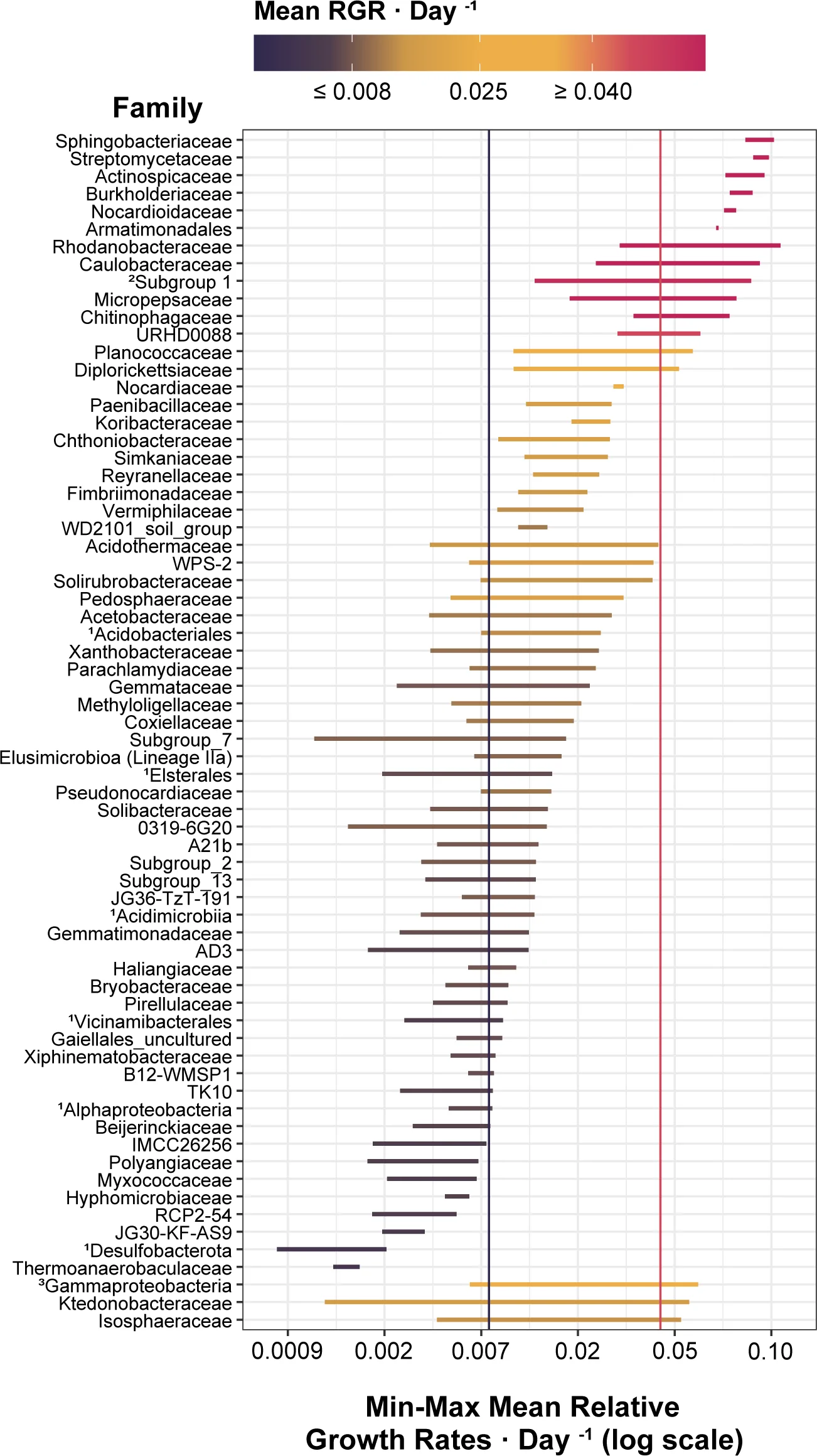
Range of taxon-specific mean RGRs by family; bars reflect the minimum and maximum values for taxa within each family (note log scale). Only families with two or more taxa were included. Color represents the mean RGR of each family. Vertical lines represent cutoff values for the trophic groups. Most families were present in one or two trophic groups and are ordered by mean RGR; however, three families had members in all three trophic groups and are listed last. Superscript 1 denotes “uncultured” family, superscript 2 represents *Acidobacteriaceae*, and superscript 3 indicates an “unclassified” family.

**Fig 4:**
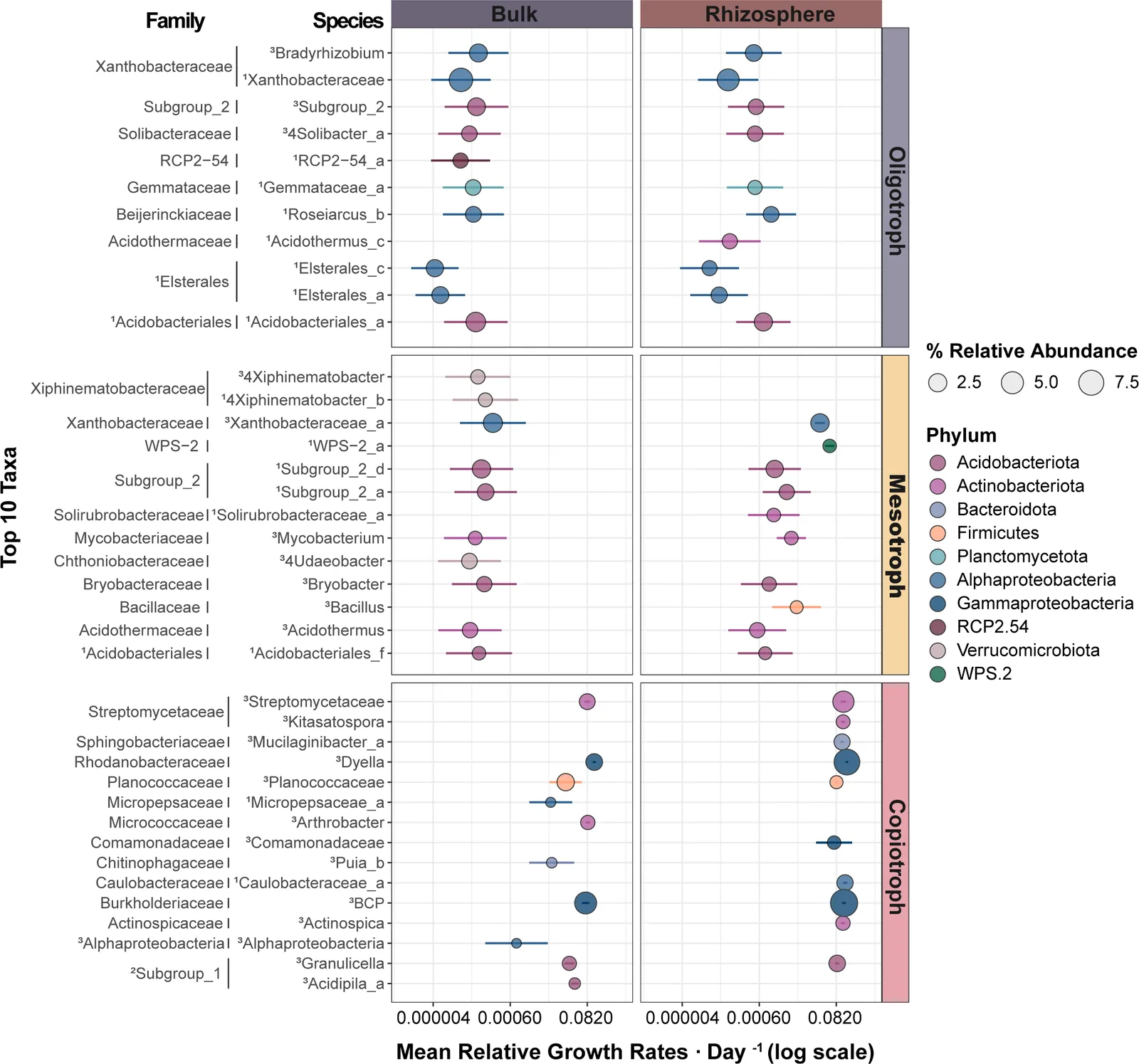
Mean (±95% confidence interval) RGRs (note the log scale) of the top 10 most abundant taxa within each trophic group and soil environment. Points are sized by relative abundance and colored by phylum. Superscript 1 = uncultured, 2 = *Acidobacteriaceae*, 3 = unclassified, and 4 = Candidatus.

Many families exhibited clear distinct trophic strategies (Fig. 3). For example, taxa within the families *Actinospicaceae*, *Burkholderiaceae, Sphingobacteriaceae*, and *Streptomycetaceae* were consistently classified as copiotrophs. Members of *Vermiphilaceae*, *Chthoniobacteraceae*, and *Simkaniaceae* were identified as mesotrophs, and the families *Polyangiaceae*, *Myxococcaceae*, and the uncharacterized RCP2-54 clade were categorized as oligotrophs. In both bulk and rhizosphere soil, taxa from *Rhizobiales*, *Elsterales*, and *Acidobacteriales* orders were found in high relative abundance within the oligotroph and mesotroph populations. Copiotrophic groups included multiple taxa within *Xanthomonadales* and *Burkholderiales*. These copiotrophic taxa (e.g., unknown *Dyella, Rhodanobacter*, *Burkholderia, Caballeronia, Paraburkholderia*, and *Comamonadaceae* species) exhibited high mean RGRs and were highly abundant in both soil habitats (Fig. 4). While members of finer taxonomic groups (e.g., families, orders) frequently exhibited consistent trophic placement, generalization could not be extended to the phylum level (Fig. S3). Large cosmopolitan phyla such as Acidobacteriota, Actinobacteriota, and Proteobacteria contained oligotrophic, mesotrophic, and copiotrophic taxa. However, less diverse phyla had members with either oligotroph and mesotroph (e.g., Verrucomicrobiota and Planctomycetota) or mesotroph and copiotroph (e.g., Firmicutes and Bacteridota) strategies (Fig. S3 and S4).

### Interactions between trophic strategy, carbon addition, and soil habitat

**Fig 5:**
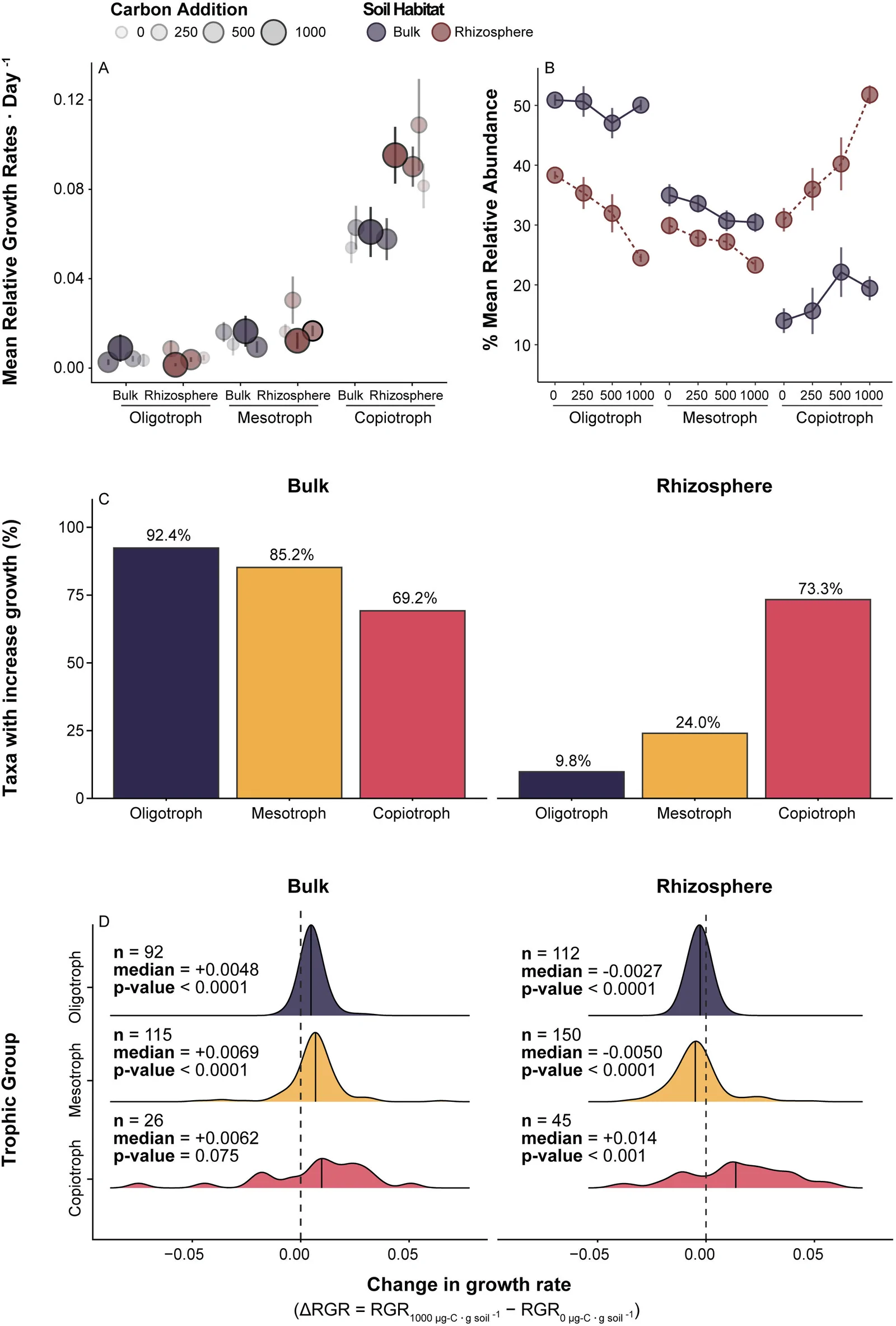
Community mean relative growth rates (**A**) and relative abundance of each trophic group (**B**) of each trophic group across soil habitats and carbon addition treatment (0, 250, 500, and 1,000 µg-C g-Soil^−1^). Values represent mean ± standard error. Changes in RGRs between 1,000 µg-C g-Soil^−1^ and 0 µg-C g-Soil^−1^ treatments, shown as the proportion of taxa exhibiting increased mean RGR in response to carbon addition for each soil habitat and trophic group (**C**). Kernel density distribution of changes in median RGR (ΔRGR = RGR_1,000 µg-C g-Soil_^_−1_^ – RGR_0 µg-C g-Soil_^_−1_^) for individual taxa within each trophic group and soil habitat (**D**). Differences in median RGR were evaluated using Wilcoxon signed-rank tests.

The effects of carbon addition and habitat on RGRs differed across the trophic strategies. While RGRs were relatively similar across soil habitats for oligotrophic and mesotrophic taxa, copiotrophs frequently had higher RGRs in the rhizosphere (Fig. 5A). The average RGR of copiotrophic taxa was 80% higher in the rhizosphere than in the bulk soil (three-way ANOVA, *F*_Soil × Strategy_ = 12.5, *P* < 0.001). Although carbon addition rarely had a statistically significant effect on taxon-specific RGRs as determined by Kruskal–Wallis (described above), carbon addition tended to increase the growth of most taxa in the bulk soil but only copiotrophs in the rhizosphere. Specifically, when comparing the 1,000 µg-C and 0 µg-C treatments, the change in taxon-specific growth was positive for the majority of oligotrophs, mesotrophs, and copiotrophs in the bulk soil (Fig. 5C and D) with the median change in RGR ranging from 0.0048 to 0.0097. In the rhizosphere, copiotrophs responded positively to carbon addition, while the RGRs of most oligotrophs and mesotrophs decreased (Fig. 5D).

The relative abundance of trophic groups was similarly impacted by carbon addition in the rhizosphere (three-way ANOVA, *F*_Soil × Exudate × Strategy_ = 3.83, *P* = 0.002) (Fig. 5B). In the bulk soil, oligotrophs constituted the largest proportion of active taxa (~50%) and carbon additions produced no significant changes in the relative abundances within each trophic group. The average (± standard error) relative abundance of oligotrophs in the bulk soil constituted approximately 50% ± 0.94% across carbon addition. While the average relative abundance of mesotrophs was 32% ± 0.90%, and copiotrophs was 18% ± 1.6% across carbon additions. In contrast, the rhizosphere soil had approximately equal proportions of oligotrophs, copiotrophs, and mesotrophs. Here, carbon addition produced a dramatic increase in the relative abundance of copiotrophs, with the largest percent increase (~67.7%) occurring between the 0 µg and 1,000 µg carbon treatments. Comparatively, the relative abundance of oligotrophs in the rhizosphere decreased with carbon addition, with the largest difference occurring between the 0 µg and 1,000 µg carbon addition groups.

## DISCUSSION

Here, we provide insight relating to soil microbial trophic strategy within the oligotrophic–copiotrophic framework using growth rate. Consistent with our hypotheses, fast-growing copiotroph taxa were more abundant in the rhizosphere and tended to have higher growth rates and increases in relative abundance following carbon addition (Fig. 5). Slower-growing oligotrophic and mesotrophic taxa were more abundant in the bulk soil, and carbon addition had variable or negative effects on RGRs and relative abundance. Furthermore, average taxon-specific bacterial growth rates were phylogenetically constrained (Fig. 3). The similarity in growth rates among closely related taxa (e.g., same family, Fig. 4), but not among distantly related taxa (e.g., same phylum), suggests that classifying microorganisms by their trophic strategies is possible at finer, but not at coarse, taxonomic levels.

While habitat and carbon additions did impact growth, taxon-specific growth rates were fairly consistent across both soil habitats and across carbon additions (Fig. S3). Namely, fast-growing copiotrophs had comparatively high growth rates, mesotrophs intermediate, and oligotrophs slow, under all conditions as indicated by robust inter-taxon variation (Table 2) and the strong covariation in growth rates across treatments (Fig. S5). Limited variation in taxon-specific growth rates likely reflects evolutionary constraints on microbial growth (Fig. 2). This is consistent with past qSIP-based studies that show growth rate to exhibit a significant phylogenetic signal (20, 53). This phylogenetic signal likely arises from evolutionary conservation of the physiological traits that determine growth rate. While the genomic basis of growth rates and trophic strategy is not fully understood, studies primarily in aquatic systems suggest genome size, metabolic potential, and transcriptional regulation vary from low to high along the oligotroph–copiotroph continuum (13, 54, 55). This genomic complexity may partially explain the observed phylogenetic signal, as complex metabolic traits tend to be better conserved (56, 57). However, this consistency in growth rate may be due at least in part to experimental conditions. Soil drying and rewetting for ^18^O addition may have increased carbon and resource availability (58), leading to sufficient available fuel for copiotrophic taxa to grow relatively quickly even in the absence of exudate addition. Moreover, because ^18^O-qSIP relies on detecting isotope incorporation into newly synthesized DNA, very slow-growing taxa may accumulate insufficient ^18^O enrichment over the incubation period to be confidently distinguished from background, potentially increasing uncertainty in growth estimates for the slowest oligotrophic taxa.

While growth rate is evolutionarily conserved, our results suggest that this conservation is not deep enough to classify entire phyla (Fig. 2). However, trophic classification may be meaningful for narrower (e.g., family and genus) taxonomic groups (Fig. 3), as previously suggested by Morrissey et al. (20) and Stone et al. (21). Lack of coherence within cosmopolitan phyla such as Proteobacteria and Actinobacteriota is not surprising, as these groups are well known to contain immense metabolic diversity (59, 60).

Taxon-specific growth rates were fairly consistent, as differences in growth rates between taxa (interspecific variation) were generally much greater than differences within taxa (intraspecific variation) across treatment groups (Table 2; Fig. S3). This aligns with previous studies showing that taxon-specific growth rates were consistent across environmental gradients (53, 61). Given that most taxa had fairly similar growth rates under various conditions, we posit that trophic strategy can be assigned using the average RGR of each taxon (Fig. 1 and 2; Fig. S3). The difference in the distribution of trophic groups between the bulk and rhizosphere soil (Fig. 5B; see Trophic Strategy and Soil Habitat section) highlights the effectiveness of using growth rates for trophic classification. We recognize that traditionally, in pure culture studies, oligotrophic and copiotrophic classification was based on the maximum growth rate (62). While pure culture conditions can be optimized to maximize the growth of individual bacterial species, it is not possible to provide ideal conditions for all taxa with a diverse natural community. Averaging RGRs across treatments allowed us to integrate variation in growth responses under different environmental conditions. This may be a more ecologically relevant and practical approach to trophic classification when using qSIP to measure growth rates in soil communities.

### Growth rate and trophic strategy

Growth rate measurements may provide a quantitative and ecologically relevant approach to characterizing bacterial trophic strategy along the oligotroph–copiotroph continuum. The approximately log-normal, slightly skewed distribution of growth rates (Fig. 1) indicates that most organisms grow relatively slowly, while a comparatively small number grow rapidly. Similar growth rate distributions have been observed in prior qSIP studies across a variety of ecosystems (27, 53, 61), suggesting that distribution-based thresholds may facilitate meaningful descriptions of trophic strategy. Contrary to the dichotomous representation of the oligotroph–copiotroph framework prevalent in the literature (12, 17, 18), our results show that many bacteria exhibit intermediate growth rates (Fig. S3; Fig. 3) and thus the scientific community may be served by the term mesotroph to describe microbes with intermediate growth rates and better represent the continuum of microbial growth.

Many of our growth rate-based trophic classifications of microbial genera agree with prior descriptions of their ecology. For instance, members of *Streptomycetaceae*, *Burkholderiaceae*, and *Sphingobacteriaceae* were identified as copiotrophs. This description is consistent with previous studies (63, 64), as members within these families are known to inhabit resource-rich environments (18) and are associated with copiotrophic traits such as fast growth rates, rapid metabolism, and high metabolic flexibility (53, 63). *Hyphomicrobiaceae*, *Acidimicrobiia IMCC26256*, *Polyangiaceae*, and RCP2-54 were classified as oligotrophs. Generally, these groups are found in carbon-poor habitats and exhibit traits associated with a slow-growing lifestyle, including efficient nutrient uptake and specialized metabolism (63, 65–67). *Chthoniobacteraceae*, *Pedosphaeraceae*, *Vermiphilaceae*, and *Simkaniaceae* may be best described as mesotrophic given their intermediate growth rates. While a few families had member taxa that spanned all three trophic strategies (e.g., *Ktedonobacteriaceae* and *Isosphaeraceae*, Fig. 2 to 5), most families had taxa belonging to one or two neighboring trophic groups. Although we describe microbial taxa as copiotrophic, mesotrophic, or oligotrophic for ease of discussion and analysis, we recognize that growth rates and trophic strategies exist along a continuum. Overall, our results show that bacterial growth rate exhibits phylogenetic organization; members of a family have similar growth rates, and as a result, many families can be meaningfully described as relatively copiotrophic, mesotrophic, or oligotrophic based on their placement along the distribution of observed growth rates.

### Trophic strategy and soil habitat

Differences in the carbon and resource environment between the bulk and rhizosphere soil have been frequently used to study microbial trophic strategy (12, 18, 68–70). Here, the rhizosphere had a higher proportion of fast-growing copiotrophic taxa, while the bulk soil had more slow-growing oligotrophic taxa (Fig. 5). This result is consistent with our first hypothesis and provides support for growth rate–based trophic strategy classification. Even in the absence of carbon addition, ~30% of taxa grew more rapidly in the rhizosphere soil, suggesting more abundant resources for growth in this habitat. The high-resource rhizosphere environment likely favors fast-growing bacteria because they can rapidly assimilate and grow using root-derived carbon (71). Indeed, fast-growing taxa typically exhibit high rates of labile carbon assimilation (61). Overall, our findings are consistent with previous studies that have observed greater proportions of putative copiotrophs in rhizosphere soil and oligotrophs in bulk soil habitats (12, 63). More abundant and active copiotrophs in the rhizosphere provide an explanation for the often observed, but poorly understood rhizosphere effects on soil biogeochemical process rates.

### Trophic strategy and carbon addition

Experimental additions of labile carbon have been used in a variety of studies to infer trophic strategy based on the hypothesis that copiotrophic taxa will respond positively, while oligotrophs will respond negatively to carbon addition (12, 63, 70). The second hypothesis inspired by this past work, that high-resource availability benefits fast-growing copiotrophs but not slow-growing oligotrophs, was only partially supported by our results. Carbon addition tended to increase growth rates for all bacteria in the bulk soil. The bulk soil is likely characterized by lower microbial biomass; our results suggest that in this less competitive environment, even slow-growing oligotrophs may be able to assimilate and benefit from carbon addition (Fig. 5B). In contrast, rhizosphere soils are more densely populated (68), and inherently dynamic carbon fluxes support the coexistence of microorganisms with different growth rates (72; Fig. 5B). The addition of labile carbon to an already carbon-rich rhizosphere may disrupt this balance by disproportionately stimulating copiotrophic taxa that are adapted for rapid exploitation of readily available substrates while reducing the relative abundance or activity of oligotrophic taxa. These shifts likely reflect fundamental physiological differences in carbon acquisition strategies, growth rates, and resource-use efficiency among microbial taxa (55, 63, 64, 73). Furthermore, the addition of carbon, on top of already abundant organic substrates in the rhizosphere, may have inhibited growth, as culture-based studies show that high substrate concentrations can reduce the growth rates of oligotrophs (30). Copiotrophs exhibited the greatest positive change in growth rate in response to carbon addition in both soil habitats (Fig. 5D), indicating that these fast-growing organisms can reliably capitalize on pulses of resource availability.

Carbon addition increased the relative abundance of copiotrophs and decreased the relative abundance of oligotrophs in the rhizosphere soil but not in the bulk soil. Given this inconsistency, changes in relative abundance following carbon addition may not be a reliable method to determine microbial trophic strategy. Indeed, many studies have found similarly variable responses (70), and classifying microbial taxa using this approach has led to conflicting results (e.g., see references 17, 22). As such, our results suggest that growth rate may be a more sensitive and reliable indicator of trophic strategy. However, our results suggest that examining changes in relative abundance alongside qSIP-based growth rate estimates can yield novel insights. For instance, copiotrophic taxa in the rhizosphere became more abundant following carbon addition, while oligotrophic taxa decreased in abundance. These changes in relative abundance likely result from differences in growth rates that manifest as changes in population size. This relative abundance shift occurred only in the rhizosphere (Fig. 5B), likely because root-derived carbon in the rhizosphere supported a larger, more metabolically active population of copiotrophs that were capable of outcompeting mesotrophs and oligotrophs for the added carbon. As carbon increased, the relative abundance of oligotrophs and mesotrophs declined (Fig. 5B), consistent with the reductions in growth rate of these taxa in the rhizosphere. In the bulk soil, members of all trophic groups exhibited increases in growth rate following carbon addition (Fig. 5), leading to relatively consistent proportions of oligotrophs, mesotrophs, and copiotrophs despite the change in activity. The concordance between qSIP-based growth rates and changes in relative abundance suggests these approaches can be used in tandem to understand the trophic strategies and ecological responses of microbial populations within natural soil communities.

### Trophic strategy and modeling microbial function

Microbial trophic strategies are now being integrated into ecosystem models like MIMICS and DEMENT. These models aim to improve prediction accuracy via considerations of microbial community composition and physiology, relative to those that condense microbial processes into a single parameter (74, 75). However, current models frequently use discrete variables to represent continuous traits and are poorly parameterized due to a lack of quality data. This work provides a blueprint for how the growth rate distributions of microorganisms can be determined for taxonomic groups, trophic groups, and whole communities, facilitating the incorporation of quantitative measures of microbial function in ecosystem models. For instance, the distinct growth rate distributions and proportions of copiotrophs, mesotrophs, and oligotrophs in rhizosphere vs bulk soils may help inform models that represent these distinct soil habitats, such as the CORPSE model (76). Integrating growth rate measurements with genomic and functional traits (e.g., body size, nutrient transport systems, rRNA copy number, or codon usage bias) may further validate our criteria for trophic classification to improve microbially explicit models of ecosystem processes.

### Conclusion

Despite rich and varied evidence for the oligotroph–copiotroph framework in aquatic ecology, we have struggled to meaningfully characterize the trophic strategies of microorganisms in soil. Our results demonstrate that 18O-qSIP microbial growth rate measurements may advance our ability to assess the trophic strategies of soil microbes. Consistent with classic predictions of the oligotroph–copiotroph framework, fast-growing copiotrophs were more abundant and active in the rhizosphere and consistently responded positively to carbon addition (Fig. 5). Slower-growing taxa (oligotrophs and mesotrophs) were generally more abundant in the bulk soil and exhibited only modest changes in growth in response to soil habitat and carbon addition. These taxa may be constrained by genetically conserved physiological traits that limit growth rates even when resources are abundant and competition is limited. Moderate phylogenetic organization in taxon-specific growth rates provides support for the vertical inheritance of traits that underlie growth rate and trophic strategies. Similar growth rates among members of families, but not phyla, suggest that narrow but not broad taxonomic groups may be meaningfully described as oligotrophs, mesotrophs, or copiotrophs. In summary, transforming the oligotroph–copiotroph framework using growth rate to identify the strategies of microbes may accelerate progress in the field of microbial ecology and lead to more accurate ecosystem models.

## Data Availability

The DNA sequence data and appropriate metadata are publicly available in the National Center for Biotechnology Information Sequence Read Archive (NCBI SRA) under BioProject accession numbers PRJNA1017906 and PRJNA1261992.

## SUPPLEMENTAL MATERIAL

Supplemental figures aem.01324-26-s0001.docx Fig. S1 to S5.

Supplemental material aem.01324-26-s0002.xlsx Table S2 and details of the root exudate cocktail.

ASM does not own the copyrights to Supplemental Material that may be linked to, or accessed through, an article. The authors have granted ASM a non-exclusive, world-wide license to publish the Supplemental Material files. Please contact the corresponding author directly for reuse.
