# Supplementary material for: Soil microbial growth rate, trophic strategy, and the oligotroph–copiotroph continuum
Source: Appl Environ Microbiol. 2026 Aug 10;92(9):e01324-26. doi: 10.1128/aem.01324-26 (PMC13599533; doi:10.1128/aem.01324-26)
Supplement: Supplementary file 1 [file aem.01324-26-s0001.docx]

**Supplementary Materials**

**Title: Soil microbial growth rate, trophic strategy, and the oligotroph-copiotroph continuum**

**Running Title**: Microbial growth rate and trophic strategy

**Authors**: Chansotheary Dang^1,2^ (dangc@vcu.edu), Juan Piñeiro Nevado^2,3^(juan.pineiron@upm.es), Ronald Schartiger^1,2^ (rgs0010@mix.wvu.edu), Binu M. Tripathi^1,2^ (binu.tripathi@mail.wvu.edu), Jeth G. Walkup^2,4^ (jwalkup@uci.edu), Edward Brzostek^1^ (erbrzostek@mix.wvu.edu), Ember M. Morrissey^1,2*^

**Affiliations**:

^1^Department of Biology, West Virginia University, Morgantown, West Virginia, USA

^2^Division of Plant and Soil Sciences, West Virginia University, Morgantown, West Virginia, USA

^3^School of Forest Engineering and Natural Resources, Polytechnic University of Madrid, Madrid, Spain

^4^Department of Ecology and Evolutionary Biology, University of California, Irvine, CA, USA

^*^**Corresponding Author**:

Email: [ember.morrissey@mail.wvu.edu](mailto:ember.morrissey@mail.wvu.edu), Phone: 304-293-5201

Present Address: West Virginia University, Department of Biology, West Virginia University, Department of Biology, Life Sciences Building, Morgantown, WV, 26501

**Keywords**: microbial growth rates, oligotroph-copiotroph continuum, life history strategy, trophic strategy, quantitative stable isotope probing, soil habitat, rhizosphere

**Data Accessibility Statement**: The DNA sequence data and appropriate metadata are publicly available in the National Center for Biotechnology Information Sequence Read Archive (NCBI SRA) BioProject: ID PRJNA1017906 and PRJNA1261992.

**Statement of Authorship**: CD, EMM, and JPN designed the experiment. CD and JPN collected soil samples. Laboratory measurements were performed by JPN and RS. CD analyzed the data and drafted the manuscript with significant contributions from all authors, JPN, RS, BMT, JGW, EB, and EMM.

**This file includes supplementary figure 1 - 5.**

**Supplementary table 2 (separate file) –** Taxon specific relative growth rate and relative abundance data.

B)

A)

**Supplementary Figure 1**: Venn diagram of the number of actively growing taxa unique to the bulk and rhizosphere environment and taxa that are found across both soil habitats (A). Differences in microbial community composition as visualized with non-metric multi-dimensional scaling (NMDS) based on Bray-Curtis dissimilarity with Hellinger transformed abundance data (B).

**Supplementary Figure 2**: Carbon mineralization rates (mean ± standard error) across soil habitat and carbon addition levels (0, 250, 500, and 1000 µg-C · g-Soil^-1^).

**Supplementary Figure 3**: Heat map of the mean relative growth rates (RGRs, n = 5) of taxa grouped by phylum across soil habitats and carbon addition levels (0, 250, 500, and 1000 µg-C · g-Soil-1). Phyla with more than three taxa are included and abundant phyla are highlighted. Grey bars indicate a taxon was not detected.

**Supplementary Figure 4**: The number of taxa per phylum grouped by trophic strategy based on mean relative growth rates (RGRs): oligotrophs (RGR ≤ 0.0073), mesotrophs (0.0073 < RGR < 0.043), and copiotrophs (RGR ≥ 0.043).

**Supplementary Figure 5**: Correlation of per-taxon growth between carbon addition levels (0, 250, 500, and 1000 µg-C · g-Soil-1), covariation is reported as spearman Rho and colored based on the strength of covariation in bulk and rhizosphere soil habitats.
